# Supplementary material for: A Comprehensive Assessment of Ultraviolet-Radiation-Induced Mutations in Flammulina filiformis Using Whole-Genome Resequencing
Source: J Fungi (Basel). 2024 Mar 20;10(3):228. doi: 10.3390/jof10030228 (PMC10971301; doi:10.3390/jof10030228)
Supplement: Supplementary file 1 [file jof-10-00228-s001.zip › Supplementary Material S8/KEGG annotation/out/64381550635650.os/KO/out_map/map04210.html]

KEGG PATHWAY: Apoptosis - Reference pathway


|  |  |
| --- | --- |
| **Apoptosis - Reference pathway** |  |

[
Pathway menu
| Organism menu
| Pathway entry
| Show description
| User data mapping
]

Apoptosis is a genetically controlled mechanisms of cell death involved in the regulation of tissue homeostasis. The 2 major pathways of apoptosis are the extrinsic (Fas and other TNFR superfamily members and ligands) and the intrinsic (mitochondria-associated) pathways, both of which are found in the cytoplasm. The extrinsic pathway is triggered by death receptor engagement, which initiates a signaling cascade mediated by caspase-8 activation. Caspase-8 both feeds directly into caspase-3 activation and stimulates the release of cytochrome c by the mitochondria. Caspase-3 activation leads to the degradation of cellular proteins necessary to maintain cell survival and integrity. The intrinsic pathway occurs when various apoptotic stimuli trigger the release of cytochrome c from the mitochondria (independently of caspase-8 activation). Cytochrome c interacts with Apaf-1 and caspase-9 to promote the activation of caspase-3. Recent studies point to the ER as a third subcellular compartment implicated in apoptotic execution. Alterations in Ca2+ homeostasis and accumulation of misfolded proteins in the ER cause ER stress. Prolonged ER stress can result in the activation of BAD and/or caspase-12, and execute apoptosis.

|  |  |
| --- | --- |
| Reference pathway | 100% |
